# Why Ortho- and Para-Hydroxy Metabolites Can Scavenge Free Radicals That the Parent Atorvastatin Cannot? Important Pharmacologic Insight from Quantum Chemistry

**DOI:** 10.3390/molecules27155036

**Published:** 2022-08-08

**Authors:** Ioan Bâldea

**Affiliations:** Theoretical Chemistry, Heidelberg University, Im Neuenheimer Feld 229, D-69120 Heidelberg, Germany; ioan.baldea@pci.uni-heidelberg.de

**Keywords:** radical-scavenging activity, atorvastatin, antioxidant mechanisms, HAT, SPLET, SET-PT, global chemical reactivity indices, DPPH radical, solvent effects, quantum chemistry

## Abstract

The pharmaceutical success of atorvastatin (ATV), a widely employed drug against the “bad” cholesterol (LDL) and cardiovascular diseases, traces back to its ability to scavenge free radicals. Unfortunately, information on its antioxidant properties is missing or unreliable. Here, we report detailed quantum chemical results for ATV and its ortho- and para-hydroxy metabolites (o-ATV, p-ATV) in the methanolic phase. They comprise global reactivity indices, bond order indices, and spin densities as well as all relevant enthalpies of reaction (bond dissociation BDE, ionization IP and electron attachment EA, proton detachment PDE and proton affinity PA, and electron transfer ETE). With these properties in hand, we can provide the first theoretical explanation of the experimental finding that, due to their free radical scavenging activity, ATV hydroxy metabolites rather than the parent ATV, have substantial inhibitory effect on LDL and the like. Surprisingly (because it is contrary to the most cases currently known), we unambiguously found that HAT (direct hydrogen atom transfer) rather than SPLET (sequential proton loss electron transfer) or SET-PT (stepwise electron transfer proton transfer) is the thermodynamically preferred pathway by which o-ATV and p-ATV in methanolic phase can scavenge DPPH${}^{•}$ (1,1-diphenyl-2-picrylhydrazyl) radicals. From a quantum chemical perspective, the ATV’s species investigated are surprising because of the nontrivial correlations between bond dissociation energies, bond lengths, bond order indices and pertaining stretching frequencies, which do not fit the framework of naive chemical intuition.

## 1. Introduction

The highly radical scavenging active cholesterol-lowering drug atorvastatin (ATV) [1] is an outstanding success sale story [2]. It was patented in 1985 and approved by the Food and Drug Administration (FDA) in 1996 for medical use. Sold under the name of Lipitor by the world’s leading pharmaceutical company Pfizer, it received record high revenues of about 12.8 billion US dollars in 2006, still generated ten billion US dollars in the year of patent loss (2011) and nearly two billion US dollars in 2019. ATV, one of the most prescribed drugs in the US today, is mainly employed to prevent high risk for developing cardiovascular diseases and as treatment for abnormal lipid levels (dyslipidemia). ATV’s inhibition of the HMG-CoA (3 hydroxy-3-methylglutaryl coenzyme A) reductase is plausibly related to the high radical scavenging potency against lipoprotein oxidation.

ATV made the object of several theoretical investigations in the past [3,4]. Still, the antioxidant properties of ATV were only recently investigated from the quantum chemical perspective [5]. Unfortunately, as we drew attention recently [6], the only quantum chemical attempt of which we are aware [5] is plagued by severe flaws [6] (e.g., “prediction” of enormous, totally unrealistic O-H bond dissociation energies of ∼400kcal/mol> 17 eV), and this makes mandatory the effort (undertaken in the present paper) of properly reconsidering the antioxidant capacity of ATV and its ortho- and para-hydroxy metabolites in methanol. For the notoriously poor soluble ATV, this solvent is of special interest. ATV is freely soluble in methanol. In addition, antioxidant assays are mostly done in methanolic environment [5,7]. Along with quantities traditionally related to the antioxidant activity, the present study will also reports on the ATV global chemical reactivity indices, relevant bond data as well as spin densities of radical species generated by H-atom abstraction from ATV and related ortho- and para-hydroxylated derivatives (o-ATV, p-ATV, respectively).

Theoretical understanding of the differences between ATV and its ortho- and para-hydroxy metabolites, which is missing to date, is of paramount practical importance. A twenty four years old experimental study reported that atorvastatin ortho- and para-hydroxy metabolites (o-ATV and p-ATV, respectively) protect, e.g., LDL from oxidation, while the parent ATV does not [8]. Importantly for the results we are going to present in Section 3.5, the free radical scavenging activity of o-ATV and p-ATV was analyzed by the ubiquitous 1,1 diphenyl-2 picryl-hydrazyl (DPPH${}^{•}$) assay in ref. [8]. Our study is able to provide the first theoretical explanation of this experimental finding.

## 2. Computational Details

The results reported below were obtained from quantum chemical calculations wherein all necessary steps (geometry optimizations, frequency calculations, and electronic energies) where conducted at the same DFT level of theory by running GAUSSIAN 16 [9] on the bwHPC platform [10]. In all cases investigated, we convinced ourselves that all frequencies are real. In all calculations we used 6-31+G(d,p) basis sets [11,12] and, unless otherwise specified (see Section 3.2 and Section 3.3), the hybrid B3LYP exchange correlation functional [13,14,15,16].

For comparative purposes, we also present results obtained by using the PBE0 [17] functional and Truhlar’s M062x [18,19] (see Section 3.2 and Section 3.3). Computations for open shell species were carried out using unrestricted spin methods (e.g., UB3LYP and UPBE0). In most radicals, employing the more computationally demanding quadratic convergence SCF methods was unavoidable. We convinced ourselves that spin contamination is not a severe issue. In all these calculations, we invariably found a value $\langle S^{2}\rangle =0.7501$ for the total spin after annihilation of the first spin contaminant, versus the exact value $\langle S^{2}\rangle =3/4$.

Still, to better check this aspect, for ATV’s cation and anion as well as for the ATV1H and ATV4H radicals (see Section 3.1 for the meaning of these acronyms) we also undertook the rare numerical effort (enormous for molecules with almost 80 atoms) of performing *full* restricted open shell (ROB3LYP) calculations; that is, not only single point calculations for electronic energy but also geometry optimization and (numerical) vibrational frequency calculations, and all these in solvent. Differences between UB3LYP and ROB3LYP were reasonably small (see Section 3.2 and Section 3.3), but they should make it clear that claims (so often formulated in the literature on antioxidation) of chemical accuracy (∼1 kcal/mol) at the B3LYP/6-31+G(d,p) are totally out of place. From experience with much smaller molecules and much simpler chemical structures (e.g., ref. [20]) we had to learn that achieving this accuracy for bond dissociation enthalpies and proton affinity (BDE and PA, quantities entering the discussion that follows) is often illusory even for extremely computationally demanding state-of-the-art compound model chemistries (CBS-QB3, CBS-APNO, G4, W1BD); see, e.g., Figure 10 of ref. [20]. DFT-calculations done by us and by others [21] revealed that, e.g., errors in ionization potential can amount up to 0.7 eV (16 kcal/mol) even when employing the functional B3LYP and the largest Pople basis set 6-311++G(3df,3pd).

Unless otherwise specified, the solvent (methanol) was accounted for within the polarized continuum model (PCM) [22] using the integral equation formalism (IEF) [23]. Although this is the “gold standard” for modeling solvents in the literature on free radical scavenging, one should be aware that this framework ignores specific solvation effects (hydrogen bonds). Because they may play an important role, e.g., in proton transfer reactions, theoretical estimates of PA may not be sufficiently accurate. While this makes comparison with experiment problematic, it should be a less critical issue when comparing among themselves PA values of various antioxidants in a given solvent (e.g., methanol). To better emphasize why we believe that solvent effects in the context of antioxidants deserve a more careful consideration, along with IEFPCM-based results, we also present results obtained in Truhlar’s SMD solvation model [24,25,26].

GABEDIT [27] was used to generate molecular geometries and spatial distributions from the GAUSSIAN output (*.log) files. To compute Wiberg bond order indices, we used the package NBO 6.0 [28] interfaced with GAUSSIAN 16. The reason why we use Wiberg bond order indices [29] rather than the heavily advertised Mayer bond order indices [30] was explained elsewhere [31]. All thermodynamic properties were calculated at T=298.15 K.

## 3. Results and Discussion

### 3.1. Molecular Geometries

**Figure 1:**
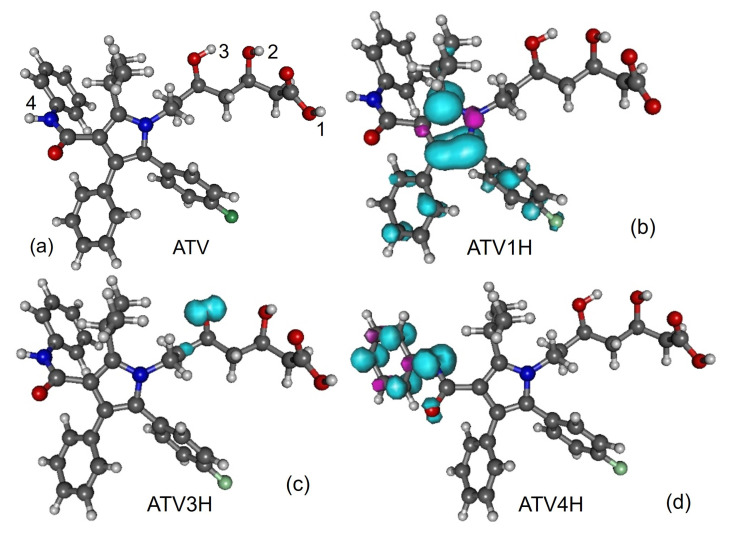
(**a**) Optimized ATV geometry. Spin densities of neutral radicals generated from it by H-atom abstraction at positions indicated in the inset: (**b**) ATV1H, (**c**) ATV3H, and (**d**) ATV4H. Figure generated using GABEDIT [27].

**Figure 2:**
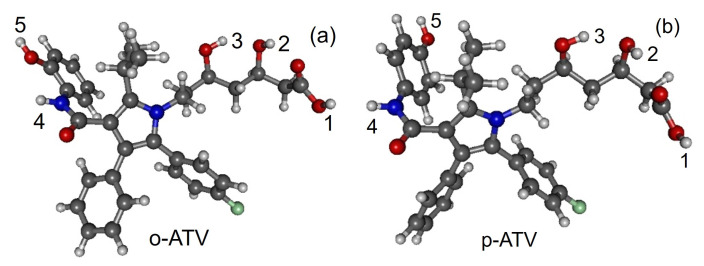
Optimized geometries of atorvastatin ortho- and para-hydroxy metabolites: (**a**) o-ATV and (**b**) p-ATV. Figure generated using GABEDIT [27].

**Figure 3:**
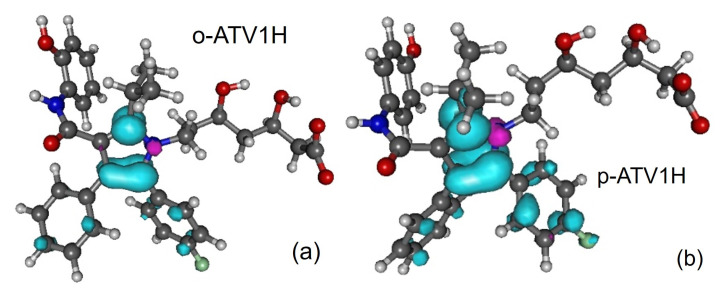
Spin densities of radicals generated by H-atom abstraction at position 1-OH: (**a**) o-ATV1H and (**b**) p-ATV1H. Figure generated using GABEDIT [27].

**Figure 4:**
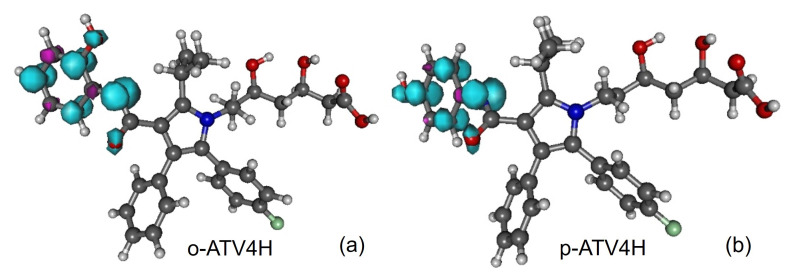
Spin densities of radicals generated from atorvastatin ortho- and para-hydroxy metabolites by H-atom abstraction at position 4-NH: (**a**) o-ATV4H and (**b**) p-ATV4H. Figure generated using GABEDIT [27].

**Figure 5:**
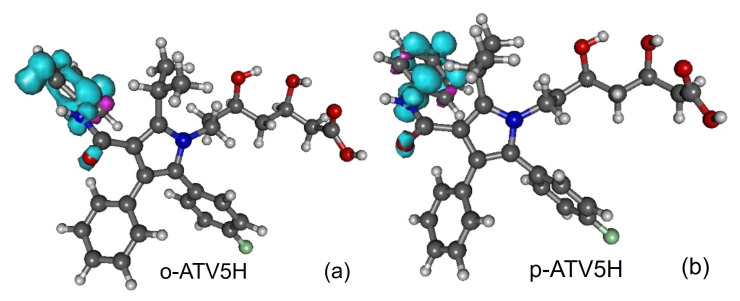
Spin densities of radicals generated from atorvastatin ortho- and para-hydroxy metabolites by H-atom abstraction at position 5-OH: (**a**) o-ATV5H and (**b**) p-ATV5H. Figure generated using GABEDIT [27].

Along with the neutral, cation and anion ATV—molecular formula C_33_H_35_FN_2_O_5_, IUPAC name (3R,5R)-7-[2-(4-fluorophenyl)-3-phenyl-4-(phenylcarbamoyl)-5-propan-2-ylpyrrol-1-yl]-3,5-dihydroxyheptanoic acid, CAS number 134523-00-5—and its metabolites ortho-hydroxy atorvastatin (o-ATV and para–hydroxyatorvastatin (p-ATV), we also investigated the radicals (e.g., ATV*n*H${}^{•}$) generated by H-atom abstraction from their O−H and N−H groups as well as the anions ATV*n*H^-^ of the latter. Here, n(=1,2,3,…) labels the various positions of the H-atoms, as depicted in Figure 1, Figure 2, Figure 3, Figure 4 and Figure 5.

All quantities to be discussed below were calculated at the total electronic energy minima of the species listed above obtained via B3LYP/6-31+G(d,p)/IEFPCM optimization (cf. Section 2), which (with the grain of salt mentioned in Section 3.4) posed no special problems. Neither H-atom abstraction (Figure 1b–d) nor ortho- and para-O−H addition (Figure 2a,b) spectacularly modifies the molecular conformation (Figure 1a). *Z*-matrices for optimized geometries of representative species are presented in Table A1, Table A2, Table A3 and Table A4 and Figure 1, Figure 3, Figure 4 and Figure 5. Rather than Cartesian coordinates, we prefer to show *Z*-matrices because they facilitate comparison between various species and methods.

### 3.2. Chemical Reactivity Indices

The global chemical reactivity indices investigated in this work are listed below along with their expressions in terms of the ionization potential IP and electroaffinity EA [32,33,34,35,36]:

(1) $$\begin{array}{cc} chemical\;hardness & \eta \equiv E_{g}/2,\\ chemical\;softness & \sigma \equiv 1/E_{g},\\ electronegativity & \chi \equiv (IP+EA)/2,\\ electrophilicity\;index & \omega \equiv \chi^{2}/\left(2\eta \right),\\ electroaccepting\;power & \omega^{+}\equiv {\left(IP+3\;EA\right)}^{2}/\left(16E_{g}\right),\\ electrodonating\;power & \omega^{-}\equiv {\left(3\;IP+EA\right)}^{2}/\left(16E_{g}\right). \end{array}$$

Here, $E_{g}\equiv IP-EA$ is the fundamental (or transport) “HOMO-LUMO” gap [32,37,38]. Noteworthily, the values of IP and EA presented in this paper were calculated as enthalpy differences (cf. Equations (4a) and (6)). Estimating IP and EA using the eigenvalues of the Kohn-Sham (KS) orbitals with reversed sign (Koopmans theorem),

(2) $$IP\to I=-E_{HOMO}^{KS};\;EA\to A=-E_{HOMO}^{KS},$$

is unfortunately a very popular approximation, but it is totally inadequate especially in the presence of a solvent. For clarification, a comment is in order at this point.

Although both the approach using Equation (2) and the approach using Equations (4a) and (6) are based on the DFT, there is an important difference between them.

Equations (4a) and (6) rely on total energies computed via DFT. In these computations, the Kohn-Sham (KS) orbitals merely enter as eigenfunctions and eigenvalues of a mathematical (minimization) problem. They are auxiliary mathematical objects useful to compute a quantity with a clear physical meaning (namely, the total electronic energy).

However, it should be well known to any well rounded theoretician that the KS “orbitals” do not have any physical meaning; they are *not* real molecular orbitals [32,38,39]. What makes Equation (2) problematic is just the fact that it treats the KS-HOMO and KS-LUMO *as if* they were the true HOMO and LUMO of a real molecule.

To remedy the difficulty related to the KS “energies” (in reality, eigenvalues of a mathematical single-particle problem) in semiconductor physics, which translates into KS-band gaps typically amounting to about 50% of the real band gap, a so-called “scissor” operator procedure is applied [40,41], which consists of empirically shifting the KS eigenvalues. To eliminate this severe difficulty in the case of molecules immersed in solvents, we also proposed a scissor technique [42]. The important difference is that the scissor corrections proposed in ref. [42] are obtained from quantum chemical calculations rather than empirically as done in semiconductor band structure calculations.

Switching back, one may expect that the global chemical reactivity indices can give a flavor of the overall stability of a molecule and are useful in predicting how a certain chemical environment evolves in time [43,44]. In certain situations they turned out to be useful for comparing properties of different molecular species [33,45,46].

**Figure 6:**
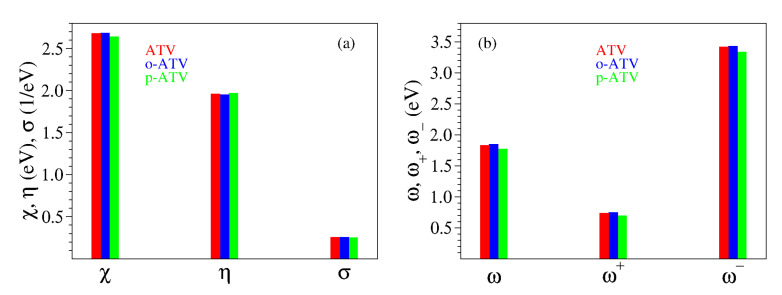
Global chemical reactivity indices defined by Equation (1) for atorvastatin and its ortho- and para-hydroxy metabolites: (**a**) electronegativity χ, chemical hardness η, and chemical softness σ; (**b**) electrophilicity index ω, electroaccepting power $\omega^{+}$, and electrodonating power $\omega^{-}$.

The presently calculated global chemical reactivity indices of ATV and its metabolites are collected in Table 1 and Table 2, and depicted in Figure 6. Having a chemical hardness η of about 2 eV, ATV, o-ATV, and p-ATV exhibit a good chemical stability. This value lies between the values of the natural antioxidants phenol and trolox, for which our calculations at the same B3LYP/6-31+G(d,p)/IEFPCM level yielded η=2.56 eV and η=1.88 eV, respectively. For all three species, the electrophilic indices [33,45,46] are ω≈1.8 eV, a value exceeding the value of 1.50 eV, which is considered the threshold for strong electrophiles [47]. For comparison, let us again mention the values ω=1.61 eV and ω=1.85 eV computed by us for phenol and trolox, respectively.

Inspection of Table 1 reveals that, similarly to the quantities η and ω considered above, all global chemical reactivity of ATV, o-ATV, and p-ATV are comparable to those of well known natural antioxidants. Could we then expect that ATV flavors (or other molecules) have indeed good antioxidant potency merely based on global chemical reactivity indices comparable to those of good antioxidants?

The analysis in the next section will unravel that, in fact, the global chemical indices have little relevance for assessing the antioxidant activity of a certain molecule. For the time being, let us remark that the values of Table 1 would rather suggest that ATV and o-ATV have similar antioxidant properties, and that ATV (possibly) performs (slightly) better than p-ATV.

### 3.3. Antioxidant Mechanisms and Pertaining Enthalpies of Reaction

As is widely discussed in the literature, an H-atom can be transferred to a free radical in one or two step processes. The three antioxidative mechanisms (HAT, SET-PT, and SPLET) and the corresponding reaction enthalpies (BDE, IP and PDE, PA and ETE, respectively) can be expressed as follows:

Direct hydrogen atom transfer (HAT) [48,49,50]

(3) $$\begin{array}{cc} AXH+R^{•}\to {AX}^{•}+RH & BDE=H\left({AX}^{•}\right)+H\left(H^{•}\right)-H\left(AXH\right). \end{array}$$

Stepwise electron transfer proton transfer (SET-PT) [51,52]

(4a) $$\begin{array}{cc} AXH\to AXH^{•+}+e^{-} & IP=H\left(AXH^{•+}\right)+H\left(e^{-}\right)-H\left(AXH\right). \end{array}$$

(4b) $$\begin{array}{cc} AXH^{•+}\to {AX}^{•}+H^{+} & PDE=H\left({AX}^{•}\right)+H\left(H^{+}\right)-H\left(AXH^{•+}\right). \end{array}$$

Sequential proton loss electron transfer (SPLET) [53,54]

(5a) $$\begin{array}{cc} AXH\to {AX}^{-}+H^{+} & PA=H\left({AX}^{-}\right)+H\left(H^{+}\right)-H\left(AXH\right) \end{array}$$

(5b) $$\begin{array}{cc} {AX}^{-}\to {AX}^{•}+e^{-} & ETE=H\left({AX}^{•}\right)+H\left(e^{-}\right)-H\left({AX}^{-}\right). \end{array}$$

In our specific case, X stands for an O or an N atom.

Related to the above (albeit not directly entering the aforementioned antioxidation mechanisms), the electron attachment process is quantified by the electroaffinity defined as

(6) $$EA=H\left(X\right)+H\left(e^{-}\right)-H\left(X^{-}\right).$$

BDE, IP, PDE, PA, and ETE are enthalpies of reaction which can be obtained as adiabatic properties from standard Δ-DFT prescriptions [38,55,56]. To this aim, along with the enthalpies of the various ATV-based species entering the above reactions, the enthalpies of the H-atom, proton and electron in methanol are also needed [6]. They are presented in Table 3.

**Figure 7:**
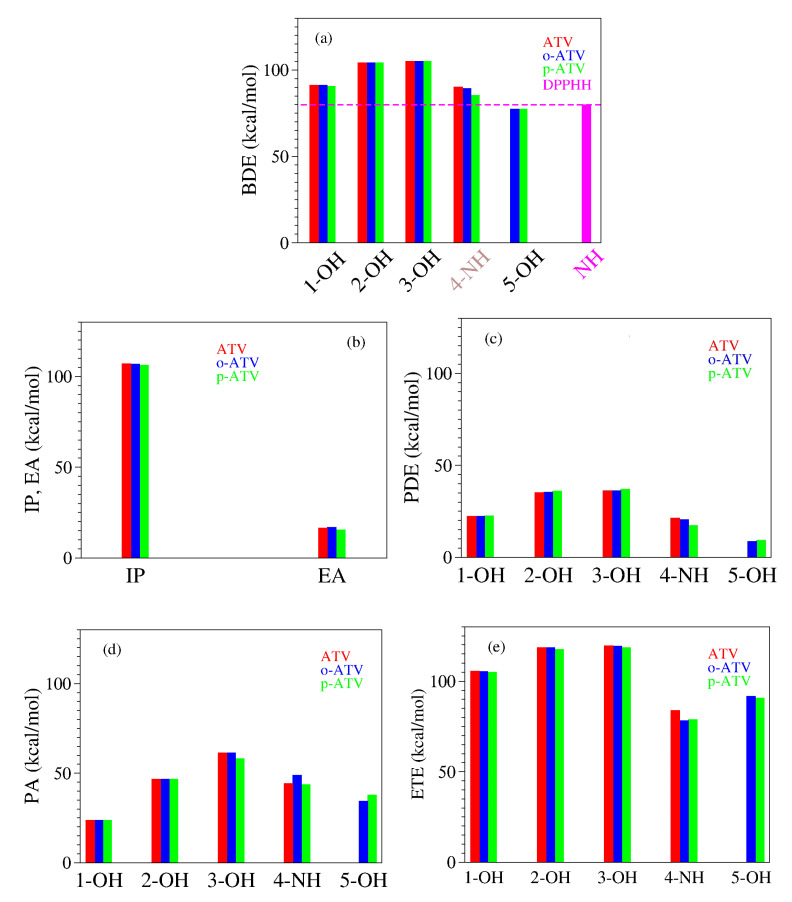
Enthalpies of reaction quantifying the antioxidant activity of atorvastatin (ATV) and its ortho- (o-ATV) and para- (p-ATV) hydroxy metabolites: (**a**) bond dissociation; (**b**) ionization and electron attachment; (**c**) proton detachment; (**d**) proton affinity; (**e**) electron transfer. The additional information for the DPPH${}^{•}$ radical in panel (**a**) depicts why o-ATV and p-ATV can scavenge this radical while the parent ATV cannot.

The presently computed thermodynamic parameters are collected in Table 4 and Table 5, and depicted in Figure 7.

Inspection of Table 4 and Figure 7 reveals that the additional 5-OH group has no notable impact on the O-H bond cleavage at positions 1-OH, 2-OH, and 3-OH, neither homolytic and heterolytic. BDE for H-atom abstraction at positions 1-OH, 2-OH, and 3-OH in ATV, o-ATV and p-ATV is basically the same. The differences between the values calculated by us for ATV, o-ATV, and p-ATV amounting to at most 0.5 kcal/mol are certainly irrelevant; recall that we showed recently [20] that even for much smaller molecules in vacuo DFT/B3LYP calculations with the largest Pople basis set 6-311++G(3df,3pd) are far away from “chemical” accuracy (∼1 kcal/mol). In fact, p-ATV’s numerical value of PA = 58.2 kcal/mol somewhat differs from ATV’s (and o-ATV’s) PA = 61.5 kcal/mol, but if heterolytic O-H bond cleavage were to occur in p-ATV, it would rather occur at position 1-OH, which has a substantially smaller value PA = 23.8 kcal/mol.

With regards to position 4-NH, the extra (5-)OH-group has a qualitatively different impact on the N-H bond cleavage of o-ATV and p-ATV. Notwithstanding the different values calculated (90.2 kcal/mol versus 89.3 kcal/mol), in the above vein we cannot soberly claim that H-atom abstraction from the NH-group is facilitated by the additional OH-group of o-ATV. However, the negative impact on the heterolytic N-H bond dissociation is significant. The o-ATV’s PA = 49 kcal/mol is larger than the value PA = 44.4 kcal/mol calculated for ATV. As of the heterolytic N-H bond dissociation, it is insensitively affected; the numerical difference between p-ATV’s PA = 43.8 kcal/mol and ATV’s PA = 44.4 kcal/mol obtained within B3LYP/6-31+G(d,p)/IEFPCM is too small to play a role in a sober analysis. Besides, similarly to what we said above, a heterolytic bond cleavage would occur at the lowest PA’s position 1-OH.

The really important effect brought about by the extra OH-group of the hydroxy metabolites is the homolytic bond dissociation at its position (5-OH). Our calculations demonstrate that this process is substantially less expensive energetically than H-atom donation from position 1-OH. The calculated BDE values for both o-ATV and p-ATV at this position are ∼77.5 kcal/mol versus the smallest value ∼91 kcal/mol for ATV at position 1-OH, respectively. Unlike the extremely similar homolytic bond dissociation, there is a certain difference between o-ATV’s and p-ATV’s heterolytic bond dissociation at position 5-OH, as expressed by the PA values (PA = 34.4 kcal/mol ≠ PA = 37.9 kcal/mol, respectively). However, it is unlikely that this difference in PA’s has practical consequences, again because the aforementioned values of PA are both comfortably larger than the lowest PA = 23.8 kcal/mol at position 1-OH, a value that also characterizes the parent ATV molecule.

In Section 3.5 we will return to the practical implications of the above finding.

### 3.4. Alternative Approaches to the O-H and N-H Bond Strengths: Vibrational Frequencies and Bond Order Indices

Let us start this section with a short digression. The robustness of a single molecule diode fabricated using the scanning transmission microscopy (STM) break-junction technique [61,62] can be quantified by the maximum force that the junction subject to mechanical stretching can withstand. This rupture (pull-off) force *F* per molecule, which characterizes the strength of the chemical bond between electrodes and the terminal (anchoring) atom of the embedded molecule, can hardly be directly measured. To circumvent this difficulty, experimentalists use a simple mechanical model which relates *F* to the vibrational frequency of the pertaining stretching mode ν. The latter quantity can be easily measured by infrared spectroscopy [63]. To exemplify, this is the Au−S stretching mode in benchmark nanojunctions wherein molecules are anchored via thiol groups on gold electrodes.

Transposed to the present context, it is interesting to interrogate the relationship between BDE and the related stretching frequency. In the same vein, a stronger chemical X-Y bond is intuitively expected to have not only a larger BDE and a higher stretching frequency ν(X-Y) but also a shorter length and a larger bond order index.

With these in mind, let us examine the correlation of the aforementioned quantities in the presently considered molecules.

**Figure 8:**
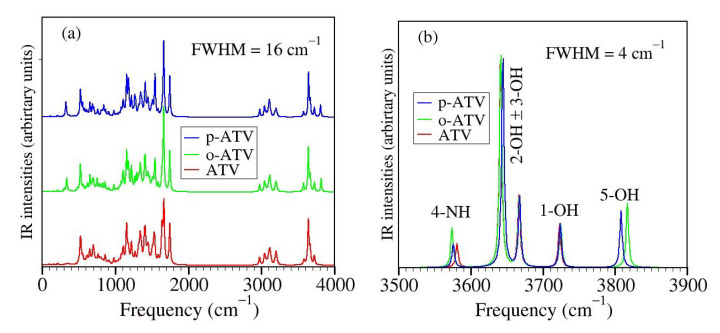
Infrared spectra calculated for ATV, o-OH-AVT, and p-ATV in methanol using Lorentzian convolution of full width at half maximum (FWHM) indicated in the inset: (**a**) in the whole range of frequency and (**b**) in the range where the O-H and N-H stretching modes are active. In all species, stretching modes of 2-OH and 3-OH groups appear as linear and antilinear vibrations rather than separated vibrational modes, and this may indicate that a more adequate optimization of the radicals generated by H-atom abstraction at these positions (which appear almost degenerate energetically, see pertaining BDE values in Table 4) should be done within a multi-reference framework.

Infrared spectra calculated for ATV, o-OH-AVT, and p-ATV in methanol are depicted in Figure 8.

The behavior visible in Figure 8b is surprising for several reasons, e.g.,

(i) although the BDE of ATV and its metabolites at position 1-OH is lower than at positions 2-OH and 3-OH, the streching mode at position 1-OH has a higher frequency than at positions 2-OH and 3-OH;
(ii) although o-ATV and p-ATV have at position 5-OH a smaller BDE than for all OH-positions of the parent ATV, the 5-OH stretching mode of the metabolites is higher than those of all O-H streching mode of ATV;
(iii) although o-ATV’s and ATV’s N-H BDE are equal, the frequency of the N-H of the former is smaller than that of the latter;
(iv) although o-ATV’s BDE and p-ATV’s BDE are different, their N-H streching modes have the same frequency;
(v) although o-ATV and p-ATV have equal BDE at position 5-OH, the o-ATV’s O-H streching frequency is higher than that of p-ATV.

**Figure 9:**
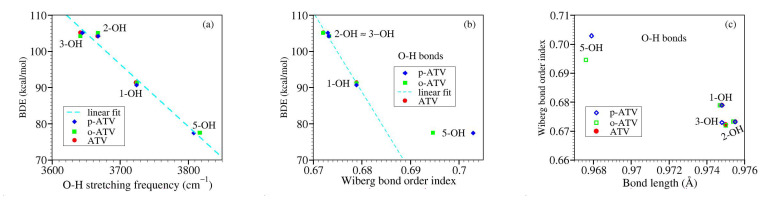
Results for OH groups of atorvastatin (ATV) and its metabolites o-ATV and p-ATV: (**a**) bond dissociation energies versus O-H stretching frequencies; (**b**) bond dissociation energies versus Wiberg bond order indices; (**c**) Wiberg bond order indices versus bond lengths.

**Figure 10:**
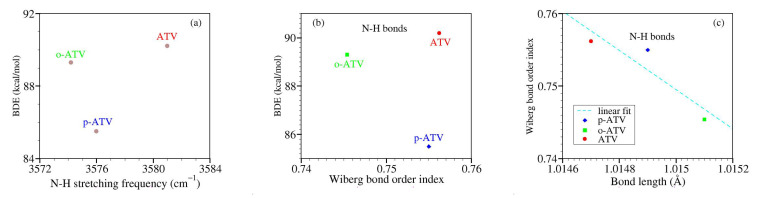
Results similar to Figure 9 but for NH groups: (**a**) bond dissociation energies versus N-H stretching frequencies; (**b**) bond dissociation energies versus Wiberg bond order indices; (**c**) Wiberg bond order indices versus bond lengths.

Counter-intuitive aspects of the relationship BDE versus ν are visualized in Figure 9a and Figure 10a.

Let us now switch to bond order indices. Our results are collected in Table 6 and Figure 9 and Figure 10.

To reiterate, based on straightforward chemical intuition, it would be obvious to expect that stronger chemical bonds (larger BDE’s) possess larger bond order indices. Figure 9b depicts that for the O-H bonds of ATV, o-ATV, and p-ATV just the opposite holds true: larger BDE’s justly correspond to smaller bond order indices. As for their N-H bonds, Figure 9b reveals that the dependence is even nonmonotonic.

To avoid misunderstanding, a clarification is in order before ending this analysis. What chemical intuition in the above example should not overlook is that a pair of atoms X and Y forming an X-Y chemical bond, do not merely interact with each other but also with the neighboring atoms in the molecular surrounding. This is also why a simple (exponential [64]) relationship between bond order indices and bond lengths can hold, e.g., for homologous molecular series [65], but cannot not hold in general; otherwise one arrives at comparing apples with oranges. Figure 9 and Figure 10 illustrate this again using the values of Table 6. BDE values corresponding to different O-H bonds of a given molecule differ from each other depending on the specific chemical environment. These differences can be visualized by inspecting the spin density landscape of the various radicals (Figure 1, Figure 3, Figure 4 and Figure 5). The stronger the delocalization in a radical, the easier is its formation, and the lower is the corresponding BDE value. Inspection of Figure 1b,c makes it clear, e.g., why ATV’s BDE at position 3-OH is higher than that at position 1-OH.

### 3.5. Assessing the Radical Scavenging Activity—A Specific Example

Discussion on free radical scavenging and dominant antioxidant mechanism is very often couched by comparing among themselves values the enthalpies characterizing the HAT, SET-PL, and SPLET of the specific antioxidant(s) under investigation. Every now and then publications conclude, e.g., that SPLET is the dominant pathway because a certain antioxidant has a “small” PA value or a PA substantially smaller than BDE, or that SET-PL prevails because of the small IP value. However, it is worth emphasizing that, along with the antioxidant’s properties, a proper evaluation of the antioxidant activity should mandatory consider the specific properties of the radicals to be eliminated (neutralized).

The small value BDE ≈77.5 kcal/mol for o-ATV and p-ATV, substantially smaller than the smallest value (BDE = 90.2 kcal/mol) of the parent ATV, is perhaps the most appealing result reported in Section 3.3. Still, the “small” value mentioned above does not demonstrate *per se* the fact anticipated in Introduction, namely that o-ATV and p-ATV can scavenge can scavenge the ubiquitously employed 1,1-diphenyl-2-picrylhydrazyl (DPPH${}^{•}$) radical, while the parent ATV cannot.

To demonstrate this, one should mandatory consider the pertaining DPPH${}^{•}$ property, namely the enthalpy release in DPPH${}^{•}$’s neutralization (H-atom affinity)

(7) $$DPPH^{•}+H^{•}\to DPPHH.$$

Because it amounts to 80 kcal/mol [66], e.g., the reaction

(8) $$o-ATV+DPPH^{•}\to o-ATV5H^{•}+DPPHH$$

is exothermic. H-atom abstraction from position 5-OH of o-ATV (or p-ATV) costs ∼77.5 kcal/mol, a value lower that the enthalpy release of 80 kcal/mol [66] in the neutralization of the DPPH${}^{•}$ radical, and this makes the HAT mechanism thermodynamically allowed. Rephrasing, because the BDE of the N−H bond of DPPHH is 80 kcal/mol [66], o-ATV (and p-ATV) can scavenge the DPPH${}^{•}$ radical through donating the H-atom at position 5-OH. On the contrary, the parent ATV cannot. The lowest ATV’s BDE (at position 1-OH) amounts to 90.2 kcal/mol (Table 4), so the HAT pathway is forbidden.

To conclude, we have presented above the first theoretical explanation of the experimental fact [8] that the antioxidant properties of atorvastatin ortho- and para-hydroxy metabolites differ from those of ATV.

By and large, there is a consensus in the literature that HAT is a possible (or even preferred) antioxidant mechanism in the gases phase but not in polar protic solvents like the presently considered methanol. In this vein, the natural question that arises is: can o-ATV and p-ATV scavenge the DPPH${}^{•}$ radical in methanol also via SPLET? Can HAT and SPLET coexist? While the large IP (Table 4) give little chances to an SET-PT pathway, SPLET would a priori be conceivable in view of the “small” value of PA, which is, although not smaller than that of ascorbic acid (as incorrectly [6] claimed in ref. [5]) at least not much larger than the latter (23.8 kcal/mol for ATV’s versus 20.5 kcal/mol for ascorbic acid, see ref. [6]).

In fact, Table 4 implicitly gives the *negative* answer to this question. If o-ATV and p-ATV could scavenge DPPH${}^{•}$ via SPLET, then (contrary to experiment [8]) the parent ATV could also do the job; the most favored deprotonation, implying the same enthalpy PA = 23.8 kcal/mol, occurs both for ATV and its metabolites at the same 1-OH position, where furthermore the similar spin density landscapes (compare Figure 1b with Figure 3) indicate a similar chemical reactivity.

Still, let us remain in the realm of theory and demonstrate why neither o-ATV nor p-ATV or ATV can scavenge DPPH${}^{•}$ in methanol via SPLET. To this aim suffice it to consider the first step of SPLET

(9) $$xATV+DPPH^{•}\to xATV1H^{-}+DPPHH^{•+},$$

where x means “o-”, “p-”, or “nothing”. Straightforward manipulation allows to express the enthalpy of this reaction as follows

(10) $$H_{r}=\underset{{PA}_{\left(xATV\right)}}{\underset{︸}{H(xATV1H)+H(H^{+})-H(xATV)}}-\underset{{PDE}_{\left(DPPHH\right)}}{\underset{︸}{H\left(DPPH^{•}\right)+H\left(H^{+}\right)-H\left(DPPHH^{•+}\right)}}.$$

Notice that the second brace in Equation (10) corresponds to the proton abstraction from the cation $DPPHH^{•+}$ of the neutralized free radical DPPHH, or alternatively, the PDE pertaining to the neutralized free radical DPPHH (cf. Equation (4b)).

Equation (10) reveals that, to be thermodynamically allowed, the first SPLET step requires

(11) PA(xATV)<PDE(DPPHH).

Our calculations yielded PDE(DPPHH)=3.9 kcal/mol, a value that is not larger (as the case if the first SPLET step was allowed) but smaller than PA(xATV)=23.8 kcal/mol. It now becomes clear why neither ATV, nor o-ATV or p-ATV can scavenge the DPPH${}^{•}$ radical via SPLET. Their “small” PA is not small enough to fulfill Equation (11).

## 4. Conclusions

We believe that the present demonstration that atorvastatin ortho- and para-hydroxy metabolites can scavenge the DPPH${}^{•}$ through donating the H-atom at the position of their extra group (5-OH), which is impossible in the parent ATV, is important not only because it theoretically explains for the first time a behavior revealed in experiment [8] but also because, from a general perspective, it provides further insight into the structure–activity relationship (SAR).

By working out a specific example (Section 3.5)—an analysis that can be straightforwardly extended to other cases—, we drew attention that an adequate approach to antioxidant’s potency should mandatory account for the thermodynamic properties of the free radicals. Equation (11) expresses a general necessary condition for thermodynamically allowed SPLET, and its application to specific cases may reveal that, even in polar solvents, free radical scavenging via this pathway is forbidden not only for ATV-based species.

In addition, our study emphasize that, while important, e.g., for modeling the temporal evolution of various molecular species interacting among themselves in a given chemical environment [65,67], the global chemical reactivity indices have no direct relevance for antioxidation. Recall that we saw in Section 3.2 that quantitative differences of ATV’s o-ATV’s, and p-ATV’s global chemical reactivity indices are minor. Furthermore, if qualitative differences in these indices were important, then, contrary to Section 3.3 and Section 3.5, o-ATV would have antioxidant properties similar to ATV rather than to p-ATV.

Last but not least, from the perspective of fundamental science, we found (Section 3.4) that properties like bond dissociation enthalpy, bond order index, bond length, and bond stretching frequency, expected after all to represent alternatives in quantifying the bond strength, are by no means correlated according to naive intuition. This finding calls for further quantum chemical efforts aiming at comprehensively characterizing ATV’s, that inherently remained beyond the scope of this study focused on ATV’s antioxidant activity. Finally, the presently reported counter-intutitve relationship between bond stretching frequency and bond strength should also be a word of caution for other communities; for example, for the molecular electronics community, wherein bond stretching frequencies (conveniently obtained via infrared spectroscopy) are used to estimate (pull-off) forces that cause the rupture of a junction subject to mechanical stretching [68].

## Figures and Tables

**Table 1:** Global chemical reactivity indices (eV) computed via B3LYP/6-31+G(d,p)/IEFPCM for atorvastatin and its ortho- and para-hydroxy metabolites and two natural oxidants in methanol.

Molecule	IP	EA	$E_{g}$	η	μ	σ	ω	$\omega^{+}$	$\omega^{-}$
ATV	4.64	0.72	3.92	1.96	−2.68	0.26	1.83	0.74	3.42
o-ATV	4.64	0.73	3.90	1.95	−2.68	0.26	1.85	0.75	3.43
p-ATV	4.60	0.67	3.93	1.97	−2.64	0.25	1.77	0.70	3.34
Phenol	5.43	0.31	5.12	2.56	−2.87	0.20	1.61	0.49	3.36
Trolox	4.51	0.76	3.76	1.88	−2.63	0.27	1.85	0.77	3.40

**Table 2:** Global chemical reactivity indices (eV) for ATV in methanol computed using 6-31+G(d,p) basis sets and the exchange-correlation functionals (B3LYP, PBE0, M062x) and solvent models (IEFPCM, SMD) specified above.

Molecule	Method	IP	EA	$E_{g}$	η	μ	σ	ω	$\omega^{+}$	$\omega^{-}$
ATV	UB3LYP/IEFPCM	4.64	0.72	3.92	1.96	−2.68	0.26	1.83	0.74	3.42
	B3LYP/SMD	4.39	0.63	3.76	1.88	−2.51	0.27	1.67	0.65	3.16
	ROB3LYP/IEFPCM	4.69	0.70	3.98	1.99	−2.69	0.25	1.82	0.72	3.42
	UPBE0/IEFPCM	4.67	0.71	3.96	1.98	−2.69	0.25	1.82	0.73	3.41
	UM062x/IEFPCM	4.95	0.73	4.22	2.11	−2.84	0.24	1.91	0.75	3.59
o-ATV	UB3LYP/IEFPCM	4.64	0.73	3.90	1.95	−2.68	0.26	1.85	0.75	3.43
	UB3LYP/SMD	4.38	0.63	3.75	1.87	−2.51	0.27	1.68	0.66	3.16
p-ATV	UB3LYP/IEFPCM	4.60	0.67	3.93	1.97	−2.64	0.25	1.77	0.70	3.34
	UB3LYP/SMD	4.37	0.61	3.77	1.88	−2.49	0.27	1.65	0.64	3.13

**Table 3:** Gas phase enthalpies $H_{0}$ and solvation enthalpies $\Delta H_{sol}$ in hartree utilized in the present calculations. For the for the gas phase enthalpy of the H-atom we used the value for the B3LYP/6-31+G(d,p) electronic energy (−0.500273 hartree) and the value of 1.4816 kcal/mol for thermal correction to enthalpy common for all compound model chemistries from GAUSSIAN 16.

Species	$H_{0}$	$\Delta H_{\mathrm{sol}}^{\mathrm{methanol}}$
Electron	+0.001194 ^a^	−0.030204 ^c^
Proton	+0.002339 ^b^	−0.405508 ^c^
H-atom	−0.497912	+0.001904 ^d^

^a^ From Ref. [57]. ^b^ From Ref. [58]. ^c^ Form Ref. [59]. ^d^ Form Ref. [60].

**Table 4:** The enthalpies of reaction (in kcal/mol) needed to quantify the antioxidant activity of atorvastatin (ATV) and its ortho- and para-hydroxy metabolites (o-ATV, p-ATV).

Molecule	Position	BDE	IP	PDE	PA	ETE
ATV	1-OH	91.4	107.0	22.4	23.8	105.7
	2-OH	104.2		35.3	46.7	118.5
	3-OH	105.2		36.3	61.5	119.5
	4-NH	90.2		21.3	44.4	83.9
o-ATV	1-OH	91.2	106.9	22.4	23.8	105.5
	2-OH	104.2		35.4	46.8	118.5
	3-OH	105.1		36.3	61.5	119.4
	4-NH	89.3		20.5	49.0	78.4
	5-OH	77.5		8.7	34.4	91.8
p-ATV	1-OH	90.7	106.2	22.6	23.8	105.0
	2-OH	104.2		36.0	46.8	117.6
	3-OH	105.1		37.0	58.2	118.5
	4-NH	85.5		17.4	43.8	79.0
	5-OH	77.4		9.2	37.9	90.8

**Table 5:** Enthalpies of reaction (in kcal/mol) computed for atorvastatin (ATV) using methods indicated above and 6-31+G(d,p) basis sets. There is no difference between unrestricted (UB3LYP) and restricted open shell (ROB3LYP) methods in calculating the PA values, and for this reason the pertaining value was written in parenthesis.

Molecule	Method	Position	BDE	IP	PDE	PA	ETE
ATV	UPBE0/IEFPCM	1-OH	93.5	107.7	23.2	24.5	106.4
	UPBE0/IEFPCM	4-OH	109.8		39.4	45.4	101.7
ATV	UB3LYP/IEFPCM	1-OH	91.4	107.0	22.4	23.8	105.7
ATV	UB3LYP/IEFPCM	4-NH	90.2		21.3	44.4	83.9
ATV	ROB3LYP/IEFPCM	1-OH	92.4	108.0	21.4	(23.8)	106.7
ATV	ROB3LYP/IEFPCM	4-NH	92.2		22.2	(44.4)	85.9
ATV	UB3LYP/SMD	1-OH	85.9	101.2	22.7	24.0	100.0
	UB3LYP/SMD	4-NH	90.7		27.6	44.0	84.8
o-ATV	UB3LYP/IEFPCM	5-OH	77.5	106.9	8.7	34.4	91.8
	UB3LYP/SMD	5-OH	79.6	101.0	16.8	34.6	83.1
p-ATV	UB3LYP/IEFPCM	5-OH	77.4	106.2	9.2	37.9	90.8
	UB3LYP/SMD	5-OH	78.0	100.9	15.2	36.5	79.5

**Table 6:** Wiberg bond order indices, bond lengths (in Å), vibrational frequencies (in cm${}^{-1}$), and bond dissociation energies BDE (in kcal/mol) for atorvastatin and its metabolites.

Molecule	Position	Wiberg	Length	BDE	ν
ATV	1-OH	0.6789	0.9748	91.4	3723.2
	2-OH	0.6732	0.9755	104.2	3667.0
	3-OH	0.6721	0.9750	105.2	3641.6
	4-NH	0.7562	1.0147	90.2	3581.0
o-ATV	1-OH	0.6789	0.9747	91.2	3724.8
	2-OH	0.6733	0.9754	104.2	3641.7
	3-OH	0.6720	0.9750	105.1	3667.5
	4-NH	0.7454	1.0151	89.3	3574.2
	5-OH	0.6946	0.9676	77.5	3817.1
p-ATV	1-OH	0.6789	0.9748	90.7	3724.2
	2-OH	0.6732	0.9755	104.2	3667.9
	3-OH	0.6730	0.9748	105.1	3644.7
	4-NH	0.7550	1.0149	85.5	3576.0
	5-OH	0.7029	0.9679	77.4	3808.4

## Data Availability

The data that support the findings of this study are available from the author upon reasonable request.

## Appendix A

**Table A1:** Z−matrix of ATV.

Atom							
F							
O	1	B1					
O	1	B2	2	A1			
N	3	B3	1	A2	2	D1	0
C	2	B4	1	A3	4	D2	0
C	3	B5	1	A4	4	D3	0
C	4	B6	3	A5	1	D4	0
C	4	B7	3	A6	1	D5	0
C	8	B8	4	A7	3	D6	0
C	6	B9	3	A8	1	D7	0
C	7	B10	4	A9	3	D8	0
C	8	B11	4	A10	3	D9	0
C	8	B12	4	A11	3	D10	0
C	12	B13	8	A12	4	D11	0
C	7	B14	4	A13	3	D12	0
H	15	B15	7	A14	4	D13	0
H	15	B16	7	A15	4	D14	0
H	15	B17	7	A16	4	D15	0
C	10	B18	6	A17	3	D16	0
H	19	B19	10	A18	6	D17	0
C	19	B20	10	A19	6	D18	0
H	21	B21	19	A20	10	D19	0
C	21	B22	19	A21	10	D20	0
H	23	B23	21	A22	19	D21	0
C	23	B24	21	A23	19	D22	0
H	25	B25	23	A24	21	D23	0
C	25	B26	23	A25	21	D24	0
H	27	B27	25	A26	23	D25	0
C	14	B28	12	A27	8	D26	0
H	29	B29	14	A28	12	D27	0
C	29	B30	14	A29	12	D28	0
H	31	B31	29	A30	14	D29	0
C	31	B32	29	A31	14	D30	0
H	33	B33	31	A32	29	D31	0
C	33	B34	31	A33	29	D32	0
H	35	B35	33	A34	31	D33	0
C	35	B36	33	A35	31	D34	0
H	37	B37	35	A36	33	D35	0
C	13	B38	8	A37	4	D36	0
H	39	B39	13	A38	8	D37	0
C	9	B40	8	A39	4	D38	0
H	41	B41	9	A40	8	D39	0
C	9	B42	8	A41	4	D40	0
H	43	B43	9	A42	8	D41	0
C	43	B44	9	A43	8	D42	0
H	45	B45	43	A44	9	D43	0
C	4	B46	3	A45	1	D44	0
H	47	B47	4	A46	3	D45	0
H	47	B48	4	A47	3	D46	0
C	47	B49	4	A48	3	D47	0
H	50	B50	47	A49	4	D48	0
H	50	B51	47	A50	4	D49	0
C	5	B52	2	A51	1	D50	0
H	53	B53	5	A52	2	D51	0
H	53	B54	5	A53	2	D52	0
O	50	B55	47	A54	4	D53	0
H	56	B56	50	A55	47	D54	0
O	53	B57	5	A56	2	D55	0
H	58	B58	53	A57	5	D56	0
O	5	B59	2	A58	1	D57	0
H	60	B60	5	A59	2	D58	0
N	6	B61	3	A60	1	D59	0
H	62	B62	6	A61	3	D60	0
C	7	B63	4	A62	3	D61	0
H	64	B64	7	A63	4	D62	0
C	64	B65	7	A64	4	D63	0
H	66	B66	64	A65	7	D64	0
H	66	B67	64	A66	7	D65	0
H	66	B68	64	A67	7	D66	0
C	47	B69	4	A68	3	D67	0
H	70	B70	47	A69	4	D68	0
H	70	B71	47	A70	4	D69	0
C	56	B72	50	A71	47	D70	0
H	73	B73	56	A72	50	D71	0
C	58	B74	53	A73	5	D72	0
H	75	B75	58	A74	53	D73	0

**Table A2:** Elements of the Z−matrix of ATV in methanol optimized as indicated below using 6-31+G/d,p) basis sets.

Element	RB3LYP	RPBE0
B1	7.63688039	7.57842858
B2	9.51376438	9.49797976
B3	4.46432846	4.45735736
B4	1.22436588	1.22105960
B5	1.24094609	1.23460721
B6	1.38313941	1.37429179
B7	1.39258446	1.38313248
B8	4.26421886	4.25026562
B9	2.55737607	2.53336720
B10	1.39352326	1.38966003
B11	1.38854352	1.38531627
B12	1.48198028	1.47533742
B13	1.47996148	1.47276739
B14	2.54735210	2.53021178
B15	1.09461863	1.09441258
B16	1.09443324	1.09451231
B17	1.09548377	1.09521443
B18	1.40411033	1.39956240
B19	1.08656542	1.08702976
B20	1.39590982	1.39231014
B21	1.08596263	1.08625533
B22	1.39827379	1.39423877
B23	1.08557774	1.08587383
B24	1.39829551	1.39470047
B25	1.08613361	1.08644255
B26	1.39684075	1.39270470
B27	1.08266424	1.08414373
B28	1.40754480	1.40342299
B29	1.08542085	1.08622867
B30	1.39703298	1.39303810
B31	1.08651573	1.08675442
B32	1.39866698	1.39488556
B33	1.08611015	1.08634947
B34	1.39859436	1.39482202
B35	1.08646176	1.08669302
B36	1.39688653	1.39286539
B37	1.08594257	1.08685598
B38	1.40608245	1.40185966
B39	1.08526008	1.08604603
B40	1.38854404	1.38594990
B41	1.08466653	1.08507152
B42	1.38886026	1.38604194
B43	1.08464963	1.08507327
B44	1.39673177	1.39264953
B45	1.08575599	1.08651262
B46	1.47041402	1.45879308
B47	1.09044919	1.09154067
B48	1.08676249	1.08820032
B49	3.14830814	3.11381339
B50	1.09708441	1.09727967
B51	1.09665251	1.09755904
B52	1.51042629	1.50242427
B53	1.09777914	1.09781828
B54	1.09295806	1.09309313
B55	2.46095421	2.44275249
B56	0.97500252	0.97301949
B57	2.45826509	2.43975864
B58	0.97546854	0.97377704
B59	1.34162105	1.33146502
B60	0.97475723	0.97138697
B61	1.37559401	1.36892180
B62	1.01471448	1.01312538
B63	1.51639092	1.50803841
B64	1.09438086	1.09568883
B65	1.54383200	1.53441635
B66	1.09274556	1.09354200
B67	1.09394603	1.09430081
B68	1.09550804	1.09534859
B69	1.53735984	1.52751574
B70	1.09529399	1.09601347
B71	1.09519081	1.09581652
B72	1.43702671	1.42351134
B73	1.10229127	1.10342439
B74	1.44374367	1.43034604
B75	1.09511016	1.09602489
A1	80.07624165	79.42212905
A2	36.81921669	36.41767374
A3	44.89960818	44.80116201
A4	69.40323071	68.51578435
A5	38.40240012	38.36009230
A6	74.54408034	74.60701401
A7	124.00299971	123.75578641
A8	142.06293646	143.13044343
A9	107.05456306	107.03652983
A10	108.31353197	108.33842834
A11	122.99231254	122.86893832
A12	126.63463731	126.55972259
A13	106.44925702	106.31547335
A14	89.06053861	89.10098490
A15	97.89435637	97.80052634
A16	142.49393445	142.62052327
A17	137.34771594	137.15532746
A18	119.50192439	119.48359463
A19	120.38662171	120.33363957
A20	119.38771650	119.39817314
A21	120.33528025	120.33582946
A22	120.40428227	120.38589644
A23	119.22951772	119.27084737
A24	120.07799529	120.08000208
A25	120.85846463	120.80411660
A26	119.79850536	119.90398307
A27	121.00661427	120.96513207
A28	119.36095172	119.32269019
A29	121.04376065	120.97477881
A30	119.57781238	119.59079132
A31	120.32876554	120.32506969
A32	120.35906970	120.34690449
A33	119.30913374	119.33935667
A34	120.13215959	120.12543035
A35	120.27437657	120.26315929
A36	119.21937981	119.25183302
A37	120.20314915	120.00645252
A38	119.42757095	119.34799906
A39	61.14506295	61.02049583
A40	120.29133823	120.18802069
A41	61.56595278	61.54130326
A42	120.28619011	120.18989970
A43	118.21356561	118.29730405
A44	118.97682184	119.03604297
A45	160.61076752	161.02034138
A46	107.85730043	108.01405941
A47	108.36720304	108.53503060
A48	155.00270587	155.09486109
A49	93.02162927	93.36411343
A50	65.83600761	65.46008045
A51	124.63027507	124.38270955
A52	106.33950994	106.35485078
A53	109.65684048	109.75541245
A54	62.50431090	62.44180079
A55	79.14840553	78.61332450
A56	94.38442272	93.89685773
A57	81.94439843	81.33655214
A58	122.30128587	122.38085758
A59	108.69599817	108.46009440
A60	118.51678659	118.96829710
A61	112.14562665	112.66775833
A62	125.70871686	125.55440414
A63	104.10605874	104.05408726
A64	115.66124171	115.41491837
A65	112.85938504	112.90944315
A66	111.09046713	111.04846793
A67	109.12827188	109.12112805
A68	112.50720874	112.20795577
A69	108.93102542	108.90860024
A70	109.76379473	109.82714850
A71	35.55295345	35.64055684
A72	108.78209604	109.15267108
A73	35.76405946	35.85622083
A74	104.88212372	105.29541039
D1	23.44707166	22.86899643
D2	−143.01621213	−145.69511584
D3	38.42108435	38.12739399
D4	167.03950592	166.71102018
D5	11.19248174	10.26295325
D6	−166.27299883	−167.50854474
D7	154.43296848	154.87100444
D8	−24.72393257	−23.90813959
D9	14.87760390	14.33966315
D10	−166.33850331	−167.39527774
D11	−178.68215577	−179.05559597
D12	116.12498790	117.52135961
D13	−96.95997467	−97.21214362
D14	10.99862661	10.70745397
D15	143.71999664	143.27578873
D16	53.45687997	56.95553385
D17	−13.05739505	−15.48602055
D18	166.77990428	164.20618170
D19	179.92648481	179.96617851
D20	−0.52972049	−0.55875352
D21	−179.66796941	−179.58911616
D22	0.73133137	0.86390555
D23	179.16217965	179.06507149
D24	0.01070889	−0.03915718
D25	179.03888636	179.05702057
D26	48.07112323	45.04719980
D27	0.74469490	0.91641539
D28	−179.96524676	−179.74189717
D29	−179.85477901	−179.85236519
D30	−0.39506461	−0.41589868
D31	−179.79735693	−179.79071017
D32	0.01649873	0.03030576
D33	179.92823773	179.93269511
D34	0.28491317	0.27631314
D35	179.18887066	179.19810092
D36	−116.75958591	−117.79096246
D37	1.86219749	1.56294201
D38	−115.86020598	−117.02116664
D39	−179.58717365	−179.74490671
D40	63.79739074	62.83877093
D41	178.95201872	179.06783274
D42	−0.28698798	−0.13593353
D43	178.22649816	178.07298377
D44	−148.93368537	−152.08506467
D45	111.42783975	113.18142815
D46	−4.05565899	−2.40606604
D47	−179.15665881	−177.51084506
D48	−12.57964549	−12.42277636
D49	−120.44409694	−120.19086848
D50	98.09202903	96.64992442
D51	91.15604637	91.57000780
D52	−153.66577605	−153.20632535
D53	135.69337038	135.76291529
D54	154.19232954	153.37488771
D55	−1.14110681	−0.79096165
D56	4.84906073	4.80879280
D57	−83.86223058	−85.19930682
D58	−1.48047919	−1.48833544
D59	161.82475625	161.81654714
D60	8.46484636	8.87965902
D61	147.77400083	149.20907105
D62	−178.10763280	−178.26760363
D63	66.16427504	65.99502020
D64	−71.70727202	−71.00674962
D65	50.12782255	50.90167682
D66	168.95273147	169.66688376
D67	−126.11657682	−124.36123085
D68	54.49143257	54.30008219
D69	−62.36290369	−62.63339700
D70	−66.30729976	−66.82133358
D71	−118.50510691	−118.64472010
D72	−128.64978340	−128.59930105
D73	−115.36964313	−115.74853701

**Table A3:** Z−matrix of ATV1H.

Atom							
F							
O	1	B1					
O	1	B2	2	A1			
N	3	B3	1	A2	2	D1	0
C	2	B4	1	A3	4	D2	0
C	3	B5	1	A4	4	D3	0
C	4	B6	3	A5	1	D4	0
C	4	B7	3	A6	1	D5	0
C	8	B8	4	A7	3	D6	0
C	6	B9	3	A8	1	D7	0
C	7	B10	4	A9	3	D8	0
C	8	B11	4	A10	3	D9	0
C	8	B12	4	A11	3	D10	0
C	12	B13	8	A12	4	D11	0
C	7	B14	4	A13	3	D12	0
H	15	B15	7	A14	4	D13	0
H	15	B16	7	A15	4	D14	0
H	15	B17	7	A16	4	D15	0
C	10	B18	6	A17	3	D16	0
H	19	B19	10	A18	6	D17	0
C	19	B20	10	A19	6	D18	0
H	21	B21	19	A20	10	D19	0
C	21	B22	19	A21	10	D20	0
H	23	B23	21	A22	19	D21	0
C	23	B24	21	A23	19	D22	0
H	25	B25	23	A24	21	D23	0
C	25	B26	23	A25	21	D24	0
H	27	B27	25	A26	23	D25	0
C	14	B28	12	A27	8	D26	0
H	29	B29	14	A28	12	D27	0
C	29	B30	14	A29	12	D28	0
H	31	B31	29	A30	14	D29	0
C	31	B32	29	A31	14	D30	0
H	33	B33	31	A32	29	D31	0
C	33	B34	31	A33	29	D32	0
H	35	B35	33	A34	31	D33	0
C	35	B36	33	A35	31	D34	0
H	37	B37	35	A36	33	D35	0
C	13	B38	8	A37	4	D36	0
H	39	B39	13	A38	8	D37	0
C	9	B40	8	A39	4	D38	0
H	41	B41	9	A40	8	D39	0
C	9	B42	8	A41	4	D40	0
H	43	B43	9	A42	8	D41	0
C	43	B44	9	A43	8	D42	0
H	45	B45	43	A44	9	D43	0
C	4	B46	3	A45	1	D44	0
H	47	B47	4	A46	3	D45	0
H	47	B48	4	A47	3	D46	0
C	5	B49	2	A48	1	D47	0
H	50	B50	5	A49	2	D48	0
H	50	B51	5	A50	2	D49	0
C	5	B52	2	A51	1	D50	0
H	53	B53	5	A52	2	D51	0
H	53	B54	5	A53	2	D52	0
O	50	B55	5	A54	2	D53	0
H	56	B56	50	A55	5	D54	0
O	53	B57	5	A56	2	D55	0
H	58	B58	53	A57	5	D56	0
O	5	B59	2	A58	1	D57	0
N	6	B60	3	A59	1	D58	0
H	61	B61	6	A60	3	D59	0
C	7	B62	4	A61	3	D60	0
H	63	B63	7	A62	4	D61	0
C	63	B64	7	A63	4	D62	0
H	65	B65	63	A64	7	D63	0
H	65	B66	63	A65	7	D64	0
H	65	B67	63	A66	7	D65	0
C	47	B68	4	A67	3	D66	0
H	69	B69	47	A68	4	D67	0
H	69	B70	47	A69	4	D68	0
C	56	B71	50	A70	5	D69	0
H	72	B72	56	A71	50	D70	0
C	58	B73	53	A72	5	D71	0
H	74	B74	58	A73	53	D72	0

**Table A4:** Elements of the Z−matrix of ATV1H in methanol optimized as indicated below using 6-31+G/d,p) basis sets.

Element	UB3LYP	ROB3LYP	UPBE0
B1	8.44235812	8.44847690	8.35899747
B2	9.48151258	9.48004054	9.44104389
B3	4.45293368	4.45205992	4.45170279
B4	1.28075664	1.28075532	1.27612637
B5	1.23516216	1.23520893	1.22884511
B6	1.39121019	1.39036552	1.38136414
B7	1.36865245	1.36927061	1.36158073
B8	4.22903226	4.22867569	4.21410980
B9	2.54297346	2.54296359	2.51827392
B10	1.41411564	1.41273105	1.41276955
B11	1.39814133	1.39906265	1.39123927
B12	1.45764082	1.45731631	1.45172243
B13	1.46214878	1.46225268	1.45692318
B14	2.52804795	2.52736311	2.50801737
B15	1.09361662	1.09358464	1.09350906
B16	1.09280828	1.09278512	1.09298184
B17	1.09400195	1.09401889	1.09384406
B18	1.40294782	1.40295766	1.39868090
B19	1.08608989	1.08608772	1.08659742
B20	1.39528664	1.39528456	1.39148253
B21	1.08563236	1.08563462	1.08591759
B22	1.39885297	1.39887769	1.39500026
B23	1.08539151	1.08539203	1.08568270
B24	1.39725830	1.39725954	1.39357881
B25	1.08569301	1.08569202	1.08600487
B26	1.39754666	1.39755038	1.39365084
B27	1.08290419	1.08290428	1.08428078
B28	1.41359961	1.41373605	1.40819345
B29	1.08383502	1.08382196	1.08499596
B30	1.39307164	1.39303645	1.38966728
B31	1.08532894	1.08532521	1.08564174
B32	1.39877845	1.39870695	1.39488367
B33	1.08554363	1.08554068	1.08589662
B34	1.40107536	1.40113592	1.39694939
B35	1.08531301	1.08531095	1.08561477
B36	1.39114277	1.39108971	1.38781466
B37	1.08454080	1.08454217	1.08578418
B38	1.41428406	1.41456146	1.40940924
B39	1.08378663	1.08375477	1.08486309
B40	1.39026744	1.39013657	1.38630505
B41	1.08392114	1.08391505	1.08433610
B42	1.39295578	1.39307149	1.39002859
B43	1.08393715	1.08393322	1.08438619
B44	1.38976598	1.38960925	1.38585905
B45	1.08386009	1.08382658	1.08487947
B46	1.48288423	1.48290265	1.46968125
B47	1.08655848	1.08656906	1.08771365
B48	1.08526299	1.08526229	1.08700526
B49	3.13186572	3.13214456	3.09809470
B50	1.09749147	1.09749242	1.09754851
B51	1.09723187	1.09723227	1.09817660
B52	1.54597249	1.54596976	1.53696411
B53	1.09826990	1.09827142	1.09817381
B54	1.09366646	1.09366220	1.09361307
B55	2.45496563	2.45493465	2.43617051
B56	0.98121613	0.98120462	0.98045950
B57	2.44581966	2.44580722	2.42582935
B58	1.00162018	1.00160987	1.00474416
B59	1.25555505	1.25555586	1.24897137
B60	1.36463937	1.36473756	1.35864347
B61	1.01542708	1.01542689	1.01391007
B62	1.50493077	1.50429573	1.49670728
B63	1.09337036	1.09333772	1.09467810
B64	1.54747843	1.54768005	1.53733187
B65	1.09151271	1.09148630	1.09246030
B66	1.09323237	1.09319168	1.09361961
B67	1.09407784	1.09408957	1.09401377
B68	1.53535621	1.53533608	1.52584909
B69	1.09518094	1.09518217	1.09596063
B70	1.09496161	1.09497326	1.09558744
B71	1.43537977	1.43540753	1.42193713
B72	1.10242221	1.10243533	1.10351859
B73	1.44492447	1.44492195	1.43098159
B74	1.09747052	1.09747351	1.09843948
A1	76.58822830	76.56600564	76.18592634
A2	36.60887483	36.62337281	36.65315300
A3	40.02401754	40.02725714	40.37741291
A4	69.22460650	69.21215273	69.11650364
A5	37.06294802	37.05454543	37.33294209
A6	75.97461715	75.97019252	75.54658203
A7	125.60763591	125.62294801	125.53862522
A8	145.46690797	145.43385778	146.47703392
A9	107.94505539	107.97228749	107.96898197
A10	108.38689995	108.36146722	108.27547546
A11	124.74777225	124.75087571	124.69207445
A12	127.97840469	127.96666547	128.15656185
A13	105.59510463	105.58982134	105.42968231
A14	89.06762423	89.07379124	89.13648017
A15	98.38552433	98.38081510	98.24507700
A16	141.42365452	141.39940160	141.62200086
A17	135.11423751	135.13907569	134.99049648
A18	119.61460474	119.61458063	119.55946866
A19	120.09632412	120.09653690	120.05115014
A20	119.42313169	119.42315027	119.43302709
A21	120.32365928	120.32410586	120.32294735
A22	120.29030763	120.28966185	120.26610493
A23	119.45834121	119.45848930	119.50076102
A24	120.18704632	120.18699066	120.19049653
A25	120.64768821	120.64769441	120.59629785
A26	119.56030742	119.56407910	119.66008477
A27	120.97200577	120.97164632	120.89552896
A28	119.95204121	119.93947727	119.96571886
A29	120.50494184	120.51489097	120.36553094
A30	119.59499068	119.60198784	119.61841115
A31	120.22405430	120.21574498	120.19645130
A32	120.10222516	120.09856981	120.06034754
A33	119.87268934	119.88010650	119.94634847
A34	120.16892282	120.16545793	120.17885990
A35	120.16243769	120.16144382	120.12026330
A36	119.28472624	119.28635927	119.38889210
A37	119.13990753	119.14065136	118.93302300
A38	119.93397869	119.91880819	119.92762403
A39	120.71429714	120.72333881	120.60124236
A40	121.46593615	121.47547577	121.48976173
A41	61.90781157	61.91592578	61.89487363
A42	120.24240110	120.24137519	120.17005220
A43	118.35259175	118.34576673	118.40770516
A44	118.86184831	118.86297431	118.93351992
A45	158.71586453	158.72856275	159.46262187
A46	107.97807868	107.98166144	108.16165610
A47	107.59850751	107.60908685	107.84756046
A48	157.42614280	157.44924436	157.60290007
A49	94.85343455	94.84153771	95.55489296
A50	64.95144615	64.95648523	64.45763585
A51	116.19161990	116.19053946	115.94779395
A52	106.86069836	106.86047108	106.91121971
A53	109.53392738	109.53458463	109.64753221
A54	62.07557911	62.08044425	61.96848271
A55	76.49524293	76.49674331	75.79460665
A56	92.76536137	92.76506052	92.30427679
A57	73.57144767	73.57345073	72.89199367
A58	125.45137451	125.45215395	125.49766270
A59	120.73260155	120.70386918	121.19251908
A60	112.50449394	112.51148946	113.02222681
A61	125.77099330	125.75901888	125.58026516
A62	104.23702836	104.25410004	104.23745904
A63	115.40821162	115.42354499	115.23936294
A64	113.24698603	113.25125527	113.34985866
A65	111.19362011	111.18145366	111.20358677
A66	107.92265897	107.90449558	107.87759561
A67	112.10461860	112.12909104	111.84315602
A68	109.13041373	109.12442468	109.15330530
A69	110.22123305	110.22217326	110.32638994
A70	35.66580947	35.66718568	35.77797455
A71	108.97338188	108.96922402	109.34397229
A72	36.23210769	36.23253122	36.38166941
A73	106.11400594	106.11371323	106.59889713
D1	20.32357302	20.28511825	19.87637559
D2	−162.17511087	−162.33220490	−162.80694109
D3	43.63016177	43.60181174	42.29185870
D4	164.26479778	164.21734263	164.45615232
D5	10.77189784	10.75274670	10.05902947
D6	−166.43746347	−166.45509545	−167.30190503
D7	158.44374263	158.23289402	158.80659445
D8	−28.08220344	−28.08496724	−26.82514391
D9	1.91266449	1.90212780	1.62776737
D10	−165.86995934	−165.89347603	−166.61940143
D11	175.37358525	175.35516221	176.13954461
D12	113.64232131	113.65660017	114.91433970
D13	−95.76535683	−95.82640318	−96.19390095
D14	12.62820769	12.57590279	12.17768961
D15	145.45934742	145.38955744	144.82678344
D16	54.19138739	54.30206745	57.11046377
D17	−17.85450383	−17.82296579	−19.82581123
D18	161.85312253	161.88684573	159.76981057
D19	179.94673200	179.94762681	179.95512591
D20	−0.60528557	−0.60218788	−0.68612039
D21	−179.59203284	−179.59336859	−179.50090877
D22	0.90546409	0.90366406	1.03940801
D23	178.93404376	178.93542059	178.83527738
D24	0.05603713	0.05403935	0.03326550
D25	177.82860566	177.83470660	177.66605428
D26	−137.16331958	−137.09552508	−138.22471461
D27	0.43265772	0.51931825	0.61777653
D28	178.15552655	178.21539172	178.43299456
D29	−178.95651780	−178.95670070	−178.99276897
D30	0.79493565	0.77240094	0.77488924
D31	179.51072096	179.53026575	179.50412377
D32	−0.68679275	−0.67915786	−0.68858323
D33	179.67314075	179.67322011	179.71709289
D34	−0.20109695	−0.20087651	−0.18063234
D35	179.30402096	179.29000187	179.32243696
D36	−130.28427078	−130.51938776	−130.58633908
D37	1.84741835	1.91470640	1.67453246
D38	179.74200519	179.74365748	179.60779307
D39	−178.45418197	−178.42099743	−178.44652258
D40	51.73180726	51.51509192	51.55090486
D41	178.78350784	178.76463222	178.80834185
D42	−0.73877044	−0.75662840	−0.65322109
D43	177.62465046	177.60834940	177.40162657
D44	−156.11290080	−156.22713857	−158.14021510
D45	116.06952548	116.07911206	117.63013987
D46	0.99099890	1.01912297	2.42551191
D47	−171.42587327	−171.42390819	−169.95676548
D48	−15.20357679	−15.21183395	−14.84818756
D49	−122.34742832	−122.36008992	−121.73630690
D50	88.06897802	87.95008988	88.85753223
D51	83.56556644	83.56187427	83.14352949
D52	−160.76875154	−160.77109993	−161.02867903
D53	132.32183428	132.32174256	132.43569018
D54	149.97524265	149.98218167	148.79908686
D55	−9.90827522	−9.91152885	−10.13040806
D56	9.74285779	9.73770241	9.81321453
D57	−93.97703409	−94.06732013	−93.02985254
D58	160.87867630	160.75346463	161.09930653
D59	6.66281917	6.73900407	7.18339300
D60	145.35128108	145.39894308	146.79423835
D61	−177.00884009	−177.06601025	−177.27755993
D62	67.06790752	66.97955721	66.63767044
D63	−71.82425328	−71.79410371	−71.15947356
D64	50.84104068	50.87713734	51.65171208
D65	169.00058929	169.03088651	169.71030546
D66	−120.64476837	−120.63362997	−119.11115232
D67	51.89044308	51.87027494	51.66320139
D68	−65.58155270	−65.59104262	−65.93846062
D69	−66.67428542	−66.66835138	−67.19000316
D70	−118.76410502	−118.75497303	−118.87135263
D71	−132.76879862	−132.76775335	−132.41059726
D72	−117.45364759	−117.45138885	−117.81963777
